# Calcium Signals in Astrocyte Microdomains, a Decade of Great Advances

**DOI:** 10.3389/fncel.2021.673433

**Published:** 2021-06-07

**Authors:** Annamaria Lia, Vanessa Jorge Henriques, Micaela Zonta, Angela Chiavegato, Giorgio Carmignoto, Marta Gómez-Gonzalo, Gabriele Losi

**Affiliations:** ^1^Neuroscience Institute, National Research Council (IN-CNR), Padua, Italy; ^2^Department of Biomedical Sciences, University of Padua, Padua, Italy

**Keywords:** astrocytes, calcium, tripartite synapses, microdomains, gliotransmission

## Abstract

The glial cells astrocytes have long been recognized as important neuron-supporting elements in brain development, homeostasis, and metabolism. After the discovery that the reciprocal communication between astrocytes and neurons is a fundamental mechanism in the modulation of neuronal synaptic communication, over the last two decades astrocytes became a hot topic in neuroscience research. Crucial to their functional interactions with neurons are the cytosolic Ca^2+^ elevations that mediate gliotransmission. Large attention has been posed to the so-called Ca^2+^microdomains, dynamic Ca^2+^ changes spatially restricted to fine astrocytic processes including perisynaptic astrocytic processes (PAPs). With presynaptic terminals and postsynaptic neuronal membranes, PAPs compose the tripartite synapse. The distinct spatial-temporal features and functional roles of astrocyte microdomain Ca^2+^ activity remain poorly defined. However, thanks to the development of genetically encoded Ca^2+^ indicators (GECIs), advanced microscopy techniques, and innovative analytical approaches, Ca^2+^ transients in astrocyte microdomains were recently studied in unprecedented detail. These events have been observed to occur much more frequently (∼50–100-fold) and dynamically than somatic Ca^2+^ elevations with mechanisms that likely involve both IP_3_-dependent and -independent pathways. Further progress aimed to clarify the complex, dynamic machinery responsible for astrocytic Ca^2+^ activity at microdomains is a crucial step in our understanding of the astrocyte role in brain function and may also reveal astrocytes as novel therapeutic targets for different brain diseases. Here, we review the most recent studies that improve our mechanistic understanding of the essential features of astrocyte Ca^2+^ microdomains.

## Introduction

Studies performed over the last two decades have revealed that astrocytes are intrinsic elements in brain circuits that through reciprocal signaling with neurons contribute to fundamental phenomena in brain function (Araque et al., 2014). Intracellular Ca^2+^ transients, which are evoked by different neurotransmitters, are fundamental for astrocyte signaling, releasing gliotransmitters (Bezzi and Volterra, 2001) that differently modulate synaptic transmission (Di Castro et al., 2011; Panatier et al., 2011), long-term synaptic plasticity (Henneberger et al., 2010; Gómez-Gonzalo et al., 2015; Vignoli et al., 2016; Papouin et al., 2017; Robin et al., 2018), cognitive functions, and behavior (Santello et al., 2019; Kofuji and Araque, 2021; Nagai et al., 2021).

Initially centered on astrocytic soma or main processes (Cornell-Bell et al., 1990; Parpura et al., 1994), early investigations then focused on small processes, revealing that Ca^2+^ elevations can either remain restricted locally or eventually propagate to the main processes and soma (Pasti et al., 1997). Localized Ca^2+^ events were first described as microdomains in Bergmann glia processes (Grosche et al., 1999). Then, microdomains were observed in different *ex vivo* and *in vivo* brain preparations, becoming the focus of several recent studies and reviews (Volterra et al., 2014; Bazargani and Attwell, 2016; Semyanov et al., 2020). A strict and shared definition of microdomains is currently missing because of the lack of knowledge of these events. Like others, we use the term microdomains to describe Ca^2+^ events that are restricted to small portions of individual astrocyte territories that can be assigned to astrocytic thin processes with high-resolution imaging techniques.

While the molecular mechanisms and physiological roles of astrocyte Ca^2+^ microdomains remain poorly defined, the development of GECIs and advanced microscopy techniques revealed a high complexity of microdomain spatial and temporal profiles (Shigetomi et al., 2013a; Srinivasan et al., 2015; Agarwal et al., 2017; Bindocci et al., 2017). Although generally much slower than neuronal Ca^2+^ events, Ca^2+^ microdomains in astrocytes may exhibit fast kinetics on a timescale similar to neurons (Stobart et al., 2018). Notably, most studies on microdomains have to face technical limitations due to the nanoscopic size and the intrinsic remarkable dynamics of these structures (Semyanov et al., 2020). These limitations include: (i) the resolution of optical microscopy, which should be ideally paralleled by super-resolution microscopy; (ii) the type and concentration of the Ca^2+^ indicator used that buffers intracellular Ca^2+^ transients; (iii) the possible photostimulation or photodamage; (iv) the use of 2D scanning as opposed to ideal 3D acquisition; (v) the type of analysis that has to consider the highly dynamic spatial-temporal profile of these events. Despite these limitations, several recent studies revealed that the intracellular Ca^2+^ activity occurring in ultra-small processes (i.e., microdomains), where “*the most important calcium transients occur*,” is crucial for the dynamic cooperation with neurons (Bazargani and Attwell, 2016). In this review, we highlight the most recent advances in the different aspects that characterize astrocyte Ca^2+^ microdomains.

## The Locus of Ca^2+^ Microdomains

Elucidating the fine anatomy of astrocytic processes is crucial for understanding how PAPs contact synapses and affect neuronal functions. Astrocyte complex morphology is mainly composed of nanoscopic irregular protrusions that are highly fluid and plastic (Bernardinelli et al., 2014; Rusakov, 2015) and account for the majority of astrocyte volume (75–85%) (Bushong et al., 2002; Bindocci et al., 2017). These structures are below the resolution of optical microscopy with smaller processes 30–50 nm in size (Rusakov, 2015). A shared nomenclature for these structures is missing, with different terms being used, including terms such as leaflets (Gavrilov et al., 2018), nodes, and shafts (Arizono et al., 2020), or more generically gliapil, in analogy with neuropil (Bindocci et al., 2017). Serial electron microscopy (EM) with 3D reconstruction (Ventura and Harris, 1999; Calì et al., 2019) revealed the intracellular organization of astrocytic organelles, while EM imaging of astrocytes after fluorescence dye-loading through a patch pipette has revealed the fine details of PAPs (Bushong et al., 2002).

The nanoscopic size of these structures is an obstacle for functional imaging of Ca^2+^ activity and innovative imaging approaches are needed (Heller and Rusakov, 2017). A recent study using 3D-super resolution microscopy combined with Ca^2+^ imaging revealed that astrocyte processes form a spongiform domain composed of a meshwork of fine structures with nodes and shafts that may form loops of reconnecting processes (Arizono et al., 2020). In line with a previous study (Shigetomi et al., 2013a), Arizono et al. (2020) found that nodes and shafts are equally present in the entire astrocyte territory irrespective of the distance from the soma. Astrocyte morphology is thus like a sponge with the meshwork of ultrathin small processes that cover the surrounding territory (Rusakov, 2015; Arizono et al., 2020), in contrast with the classic view of star shaped cells. Analysis of the spatial relationship with excitatory synapses revealed that most PAPs are nodes, i.e., compartmentalized structures (0.07–0.7 μm^2^), where the majority of localized Ca^2+^ transients occur and may or may not propagate to nearby shafts. A strict correlation was reported between node and spine size, and also between Ca^2+^ transients and spine size (Arizono et al., 2020), suggesting that PAPs are morphologically and functionally correlated with their synaptic partners. Accordingly, nodes would be the “primary site” of Ca^2+^ signaling in excitatory tripartite synapses and may represent specialized compartments endowed with all the machinery needed for governing astrocyte-neuron dynamic interactions. Of note, a significant portion of spines is in contact with shafts (35% compared to 55% contacting nodes), which could differ in terms of Ca^2+^ sources and handling from nodes.

## The Genesis of Ca^2+^ Microdomains

Calcium transients at astrocyte soma and main processes have been studied by conventional bulk-loading with Ca^2+^-sensitive membrane-permeable chemical dyes. This approach is, however, inadequate for revealing Ca^2+^ changes at fine PAPs. The only available, though invasive, tool to study Ca^2+^ signaling at PAPs was the microinjection of chemical dyes into individual astrocytes through glass pipettes (Rungta et al., 2016). However, GECIs recently emerged as a powerful tool to study Ca^2+^ dynamics also in astrocytic microdomains (Shigetomi et al., 2013a; Kanemaru et al., 2014). The release of Ca^2+^ from the endoplasmic reticulum (ER) into the cytosol can be initiated by stimulation of metabotropic G_*q*_ G-protein-coupled receptors (GPCRs) through activation of PLC, production of IP_3_, and activation of IP_3_ receptors (IP_3_Rs) on ER membranes (Perea et al., 2009). Notably, G_*i*_ coupled GPCRs, such as GABA_*B*_ receptors, can induce Ca^2+^ transients in astrocytes through an intracellular pathway that seems to converge also on IP_3_Rs signaling (Mariotti et al., 2016, 2018; Durkee et al., 2019; Caudal et al., 2020).

Historically, PAPs have been considered structures without organelles. However, through the use of serial block-face scanning EM, one or more subcellular organelles, including ER and mitochondria, were detected in about 40% of cortical PAPs (Aboufares El Alaoui et al., 2021). Consistent with the presence of ER in PAPs, deletion of the IP_3_R type 2 (IP_3_R2), which is known to be enriched in astrocytes, partially reduces the number or amplitude (∼60–70% of reduction) of spontaneous Ca^2+^ microdomains (Kanemaru et al., 2014; Srinivasan et al., 2015; Stobart et al., 2016; Agarwal et al., 2017, but see Rungta et al., 2016). In these mice, the level of impairment of Ca^2+^ microdomain responses to different GPCR agonists or electrical stimuli is, instead, variable among different studies (Kanemaru et al., 2014; Srinivasan et al., 2015; Stobart et al., 2016; Agarwal et al., 2017). However, the IP_3_R2 seems not to be the only astrocytic receptor mediating Ca^2+^ release from the ER. Okubo et al. recently used the ER luminal Ca^2+^ indicator G-CEPIA1*er* to visualize spontaneous and evoked ER Ca^2+^ release in astrocytes. While in fine processes of wild-type (WT) mice they observed spontaneous Ca^2+^ elevations with cytosolic GCaMP6f and spontaneous ER Ca^2+^ release with G-CEPIA1*er*, in IP_3_R2^–/–^ mice they could detect spontaneous cytosolic Ca^2+^ elevations without decreases in ER Ca^2+^ levels. In contrast, in fine processes of IP_3_R2^–/–^ mice, Gq receptor activation evoked a significant Ca^2+^ release from ER, although attenuated with respect to WT. Interestingly, this IP_3_R2-independent Ca^2+^ release induced low cytosolic Ca^2+^ elevations but robust Ca^2+^ transients in mitochondria (Okubo et al., 2019). Accordingly, in IP_3_R2^–/–^ mice, IP_3_R1/3 and/or ryanodine receptors (Parpura et al., 2011; Sherwood et al., 2017) possibly account for residual IP_3_R2-independent Ca^2+^ release. Because evidence of ER cisternae is present in only ∼45% of PAPs, other ER-independent mechanisms must occur to allow Ca^2+^ dynamics in ER-free PAPs (Aboufares El Alaoui et al., 2021). For instance, Bergles’s laboratory has recently described that mitochondria are important players in astrocytic Ca^2+^ microdomains, in WT and IP_3_R2^–/–^ mice. The authors demonstrated that Ca^2+^ microdomains can arise through Ca^2+^ efflux from mitochondria after transient openings of the mitochondrial permeability transition pore (mPTP). The opening of mPTP is facilitated by the production of reactive oxygen species owing to a rise in the metabolic demand after increased neuronal activity, suggesting a direct link between metabolic rate and Ca^2+^ microdomains (Agarwal et al., 2017). Although Ca^2+^ microdomains rely in part on Ca^2+^ release from intracellular stores, they may depend also on transmembrane Ca^2+^ influxes (Rungta et al., 2016). The substantial contribution of Ca^2+^ influxes from the extracellular space to spontaneous Ca^2+^ transients in PAPs has been established with Ca^2+^-free buffers (Srinivasan et al., 2015; Rungta et al., 2016; Gómez-Gonzalo et al., 2018).

Plasma membrane channels and transporters have been proposed to contribute to Ca^2+^ microdomain activity. Among these, the involvement of the TRPA1 channel has been described (Shigetomi et al., 2012, 2013b), though a clear role of this channel has not been conclusively established (Rungta et al., 2016). A promising candidate to directly link neuronal activity with Ca^2+^ microdomains is the Na^+^/Ca^2+^ exchanger (NCX). Na^+^-dependent neurotransmitter transporters localized in PAPs, such as the GABA transporter GAT3 (Minelli et al., 1996) or the glutamate transporter GLT-1 (Danbolt et al., 1992; Sakers et al., 2017), can generate Na^+^ microdomains (Felix et al., 2020) which in turn can trigger Ca^2+^ elevations in PAPs through the reverse function of the NCX, as recently described in the hippocampus after exogenous GABA applications (Boddum et al., 2016). However, the role of this mechanism in Ca^2+^ microdomains, both spontaneous and evoked, has been poorly investigated (Matos et al., 2018).

Although Ca^2+^ microdomains can have a pure intracellular or extracellular Ca^2+^ source, a cooperativity that finely shapes Ca^2+^ transients may occur among different sources. The release of Ca^2+^ from the ER typically triggers activation of store-operated Ca^2+^ entry (SOCE). During SOCE, depletion of ER Ca^2+^ stores activates the Ca^2+^ sensor stromal interaction molecules (STIMs) to translocate to the junctional ER-plasmatic membranes, where STIMs interact with and activate the store-operated Ca^2+^-release-activated Ca^2+^ channels (CRAC channels) formed by Orai proteins. Recently, Toth et al. (2019) observed that the frequency and amplitude of Ca^2+^ transients evoked by thrombin in PAPs from Orai1^–/–^ mice were substantially attenuated, suggesting that Ca^2+^ microdomains rely on coordinated Ca^2+^ release from intracellular stores and transmembrane Ca^2+^ influxes from the extracellular space.

**FIGURE 1:**
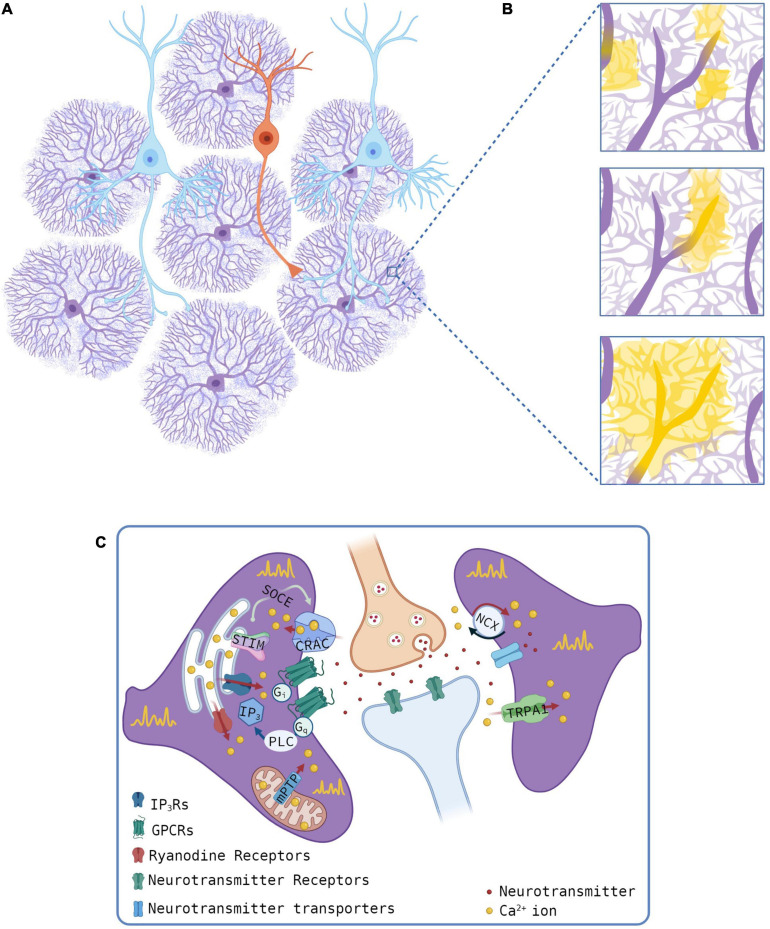
Sources of Ca^2+^ transients at microdomains. **(A,B)** Schematic of astrocyte-neuron network (**A**, pyramidal neurons, cyan, and interneuron, orange) and microdomain Ca^2+^ events **(B)**. Yellow regions for different Ca^2+^ events, as observed with optical microscopy, and the presumed thin processes (light purple) as revealed by super-resolution imaging studies. **(C)** Mechanisms of astrocyte Ca^2+^ transients at microdomains showing intracellular (left) or extracellular (right) Ca^2+^ sources. Gliotransmitters are omitted. Created with BioRender.com.

Even though knowledge is rapidly increasing (Figure 1), a complete picture of the Ca^2+^ microdomain essential features is lacking. Key questions remain unsolved. For example, which proteins are involved in Ca^2+^ influxes shaping microdomains? Astrocytes are mechanosensitive cells and the high motility of astrocyte processes (Bernardinelli et al., 2014) may expose them to physical forces that, in turn, may activate Ca^2+^-permeable mechanosensitive channels. Some plausible candidates that mediate mechanically induced Ca^2+^ microdomains have been described in astrocyte cultures and include TRPC1 (Reyes et al., 2013), TRPV4 (Turovsky et al., 2020), and Piezo1 (Velasco-Estevez et al., 2020) channels. Furthermore, are Ca^2+^ influxes modulated by activation of GPCRs? In addition to the IP_3_R-dependent signaling, GPCRs trigger different second messenger pathways, such as DAG-PKC and cAMP-PKA, that may regulate proteins mediating Ca^2+^ events.

The intracellular signaling mechanism that links activation of GPCRs to an increase in Ca^2+^ microdomains in IP_3_R2^–/–^ mice is also undefined. A partially unaltered neuronal function and the lack of somatic Ca^2+^ elevations in IP_3_R2^–/–^ mice have been used to cast doubts about the role of astrocytic Ca^2+^ signaling in brain function (Fiacco and McCarthy, 2018). The observation of a richer Ca^2+^ activity at microdomains than at soma and the relative preservation of evoked Ca^2+^ microdomains in IP_3_R2^–/–^ mice have turned attention to the relevance of astrocytic Ca^2+^ signaling in the modulation of neuronal function (Bazargani and Attwell, 2016; Savtchouk and Volterra, 2018). However, further evidence on the mechanisms of evoked Ca^2+^ microdomains in IP_3_R2^–/–^ mice is necessary to unambiguously support a role for IP_3_-independent astrocytic Ca^2+^ signaling in neuronal function. The development of new tools to impair astrocytic Ca^2+^ signaling in an IP_3_R-independent manner (Yu et al., 2018) will help to better understand the molecular mechanism of Ca^2+^ microdomain generation.

## The Analysis of Ca^2+^ Signals in Astrocytic Microdomains

Many experimental and analytical approaches have been developed to properly dissect the features of Ca^2+^ signals at the level of astrocytic microdomains. The measurement of Ca^2+^ fluctuations in astrocytic soma is relatively simple, owing to the easy identification of the somatic area. Analysis of Ca^2+^ signals in astrocytic microdomains, instead, is complex because their morphological features cannot be accurately defined under routinely used confocal or 2-photon microscopy techniques. For this reason, their identification has been handled based on the detection of Ca^2+^ changes rather than on morphological criteria. We can, indeed, gather information on microdomain Ca^2+^ signaling by imaging the fluorescent Ca^2+^ signal without, however, obtaining indications of fine process morphological details. The fluorophores of choice for this purpose have been the cytosolic or sub-membrane GECIs of the GCaMP family which revealed unexpectedly a high number of localized Ca^2+^ events throughout astrocyte distal territories (Shigetomi et al., 2013b) albeit without an estimate of the actual Ca^2+^ concentration. The increased complexity of Ca^2+^ signals revealed by GECIs, however, demanded a corresponding breakthrough in the analytical approach to properly evaluate the Ca^2+^ events. In this context, Khakh’s laboratory performed pioneering work in the decoding of astrocytic Ca^2+^ activity at the level of microdomains. Published in 2015, GECIquant represents the first software written to specifically analyze Ca^2+^ transients in GCaMP expressing astrocytes. Using a threshold-based criterion for signal amplitude, GECIquant obtains a semi-automated detection of the regions of interest (ROIs) containing Ca^2+^ fluctuations (Srinivasan et al., 2015). The software works at single cell level allowing us to distinguish between somatic Ca^2+^ fluctuations, waves involving principal processes, and sparkly signals at microdomains, providing as principal output, the raw fluorescence data along time from each region. Traces obtained through GECIquant can be further processed to obtain additional features, such as the amplitude and frequency of the Ca^2+^ events for each ROI, as performed in Mariotti et al. (2018), where a MATLAB script was used to identify significant peaks, based on additional amplitude thresholds along global and local baselines (Mariotti et al., 2018).

In 2017, CaSCaDe software was developed, overcoming the need for two scripts (Agarwal et al., 2017). CaSCaDe, like GECIquant, performs a semi-automated analysis, in this case using a machine-learning-based algorithm to identify Ca^2+^ microdomains. Time-series images are processed to remove background noise and binarized using an amplitude threshold. Microdomains are selected based on additional criteria of amplitude and duration. Besides producing a spatial map of active regions, the software provides information about the number, frequency, amplitude, and time course of events.

Weber’s laboratory worked to develop a processing suite aimed at simplifying the analysis workflows. This is the main purpose of CHIPS, a MATLAB based open-source toolbox that gathers different algorithms and provides the user with options for a number of analysis types. CHIPS offers helpful pre-processing functions, such as motion correction and denoising. Different algorithms can then be applied to analyze data and plot the resulting outputs (Barrett et al., 2018).

All the analytical procedures described so far rely on the concept of ROIs applied to microdomains, defined by spatial coordinates and measurable along different time series. However, constraining microdomains in defined boundaries may result in signal detection inaccuracy. Regions involved in Ca^2+^ variations may become larger than the defined ROI, leading the signal to invade other territories, finally resulting in signal fragmentation among adjacent ROIs. Conversely, larger ROIs may fail to faithfully capture the signal amplitude if Ca^2+^ increases only in a portion of the ROI.

The turning point in this concern was represented by AQuA software (Wang et al., 2019), which reverses the logic at the basis of ROI detection by adopting an event-based analytical approach, as already suggested in Wu et al. (2014). Calcium events are thus captured in their dynamic changes along space and time, revealing signals that can propagate and modify their size or shape, expressing different tracts of the same, composite fingerprint.

AQuA software does not include pre-processing functions but offers suggestions about pre-processing steps to be considered. A valuable feature is the possibility to choose between different data type pre-sets, depending on the Ca^2+^ indicator used and on the acquisition signal to noise ratio, with optimized but user-adjustable parameters. AQuA can work also on a single cell level and isolate events in astrocytic processes, but without distinction between principal branches and thin processes. The identification of events is achieved through different image processing steps, including thresholding and smoothing, and the application of a number of algorithms that aim to define the temporal limits and spatial propagation features of individual events. The user proceeds step-by-step and can inspect traces and spatial boundaries of the events along time, as training which is recommended to achieve the best parameters to apply to the specific experimental datasets. AQuA outputs a broad and rich collection of features for detected events, such as area, amplitude, duration, frequency, and propagation features, revealing itself as a valuable tool for astrocytic Ca^2+^ analysis.

While the approaches described so far refer to 2D imaging, it is worth mentioning that a study from Volterra’s group focused on the analysis of astrocytic Ca^2+^ activity recorded through 3D imaging. This approach captures the entire extension of individual astrocytes, adding a significant step of complexity to the comprehension of Ca^2+^ signal dynamics (Bindocci et al., 2017).

All in all, the development of different analytical approaches confirms the important role acquired by astrocytes in the studies of brain function. In the near future, analytical and experimental advances will enable us to unveil new types of modulation exerted by astrocytes.

## Discussion

Over the last decade, the combined use of innovative tools has provided compelling evidence that astrocyte dynamic interactions with neurons have fundamental roles in brain function. The different players involved in this interplay are beginning to be elucidated, particularly regarding Ca^2+^ activities at microdomains. In this restricted and elusive subcellular region, an unexpected complexity has emerged. Here, the stage of Ca^2+^ activity has been populated by new actors but many questions remain unanswered. For example, how these players interact? How Ca^2+^ events propagate from fine to major processes? What is the role of spontaneous microdomain Ca^2+^ activity in brain functions? The use of different types of preparations, Ca^2+^ detection techniques, and analytical tools in different brain regions has provided invaluable results that, however, cannot always be easily compared. New shared techniques and analytical tools are thus needed. Elucidating all the mechanisms of neuron-astrocyte interplay at tripartite synapses is crucial to understanding brain functions and dysfunctions (Pekny et al., 2016), and will possibly unveil new therapeutic targets. We believe that a new decade of great discoveries has just begun.

## Author Contributions

AL wrote the introduction with GL and the chapter on the Analysis of Ca^2+^ events with MZ. VH created the figure. AC wrote the chapter on the locus of Ca^2+^ with GL. MZ wrote chapter on the Analysis of Ca^2+^ events with AL. GC contributed to the manuscript. MG-G wrote the chapter on the genesis of Ca^2+^ events. GL wrote the abstract, introduction, the chapter on the site of Ca^2+^ events with AC, and the discussion with the help of all other authors. All authors contributed to the article and approved the submitted version.

## Conflict of Interest

The authors declare that the research was conducted in the absence of any commercial or financial relationships that could be construed as a potential conflict of interest.
